# A Clergyman’s Congregation

**Published:** 1882-04

**Authors:** 


### A CLERGYMAN'S CONGREGATION.

The following anecdote is related as having actually occurred not many months ago in a large northern seaport city in England, and we have no reason to disbelieve it.

It was a Sunday, and it was raining as it never does rain but in the vicinity of mercantile shipping on the first day of the week. The docks boasted a little church or bethel, which hoisted the union jack every Sunday morning in token that service would be held there, chiefly for sailors. The clergyman who officiated weekly at the bethel was rather later than usual on the Sunday morning in question, owing to the difficulty he had in getting a cab, the rain having caused those vehicles to be in great demand. He arrived, however, a few minutes before eleven, and hurriedly bidding the driver wait for him till service should be over, he entered the sacred edifice—to find himself alone there. The clergyman was a zealous man, so he resolved to wait a quarter of an hour, on the chance of some waif or stray turning up. His patience was not unrewarded, for after the lapse of a few minutes one very wet man came slowly in, and seated himself with some hesitation on one of the back benches. Now, our parson was not only a zealous but a conscientious man —not always the same thing—and he resolved that had he but one solitary unit instead of a congregation, he would pursue the service in full to the bitter end for that unit's benefit—at least, as long as the unit would bear it—and he proceeded to do so, and accomplished it. At the end of the liturgy, touched probably by the patient endurance of his auditor, he condescended to address him personally, telling him that since the inclemency of the weather had prevented the usual attendance at the church, he would forego the sermon he had prepared, and would content himself with making a "few remarks." This, however, his hearer begged him not to do, and expressed a great desire to hear the sermon; so, pleased with this evidence of intelligence among the lower orders, and gratified by the effect his eloquence was producing, he took the victim at his word, and let him have it. The text duly chosen, blossomed into firstly, secondly, thirdly, fourthly and lastly. "In conclusion" was followed by "one word more," and still the unit sat on undismayed.

After it was all over the preacher came down and shook hands with him, thanking him warmly for his attention, his gratification being somewhat diminished when he discovered the enraptured listener to be his cabman, the sum total of whose "half a crown an hour for waiting" had been materially augmented by the length of the worthy divine's discourse.—*Chambers' Journal.*

---

PERSIA now offers as promising a field for soldiers of fortune as Russia a century ago. Not a few Germans and Poles hold high posts in the Shah's army. The flying column recently sent against the invading Kurds contained five Austrian officers, and a Frenchman, M. Vouvilliers, has for several years held the position of chief of the arsenal at Teheran. The history of mediæval

Persia abounds in similar cases, the most remarkable of which is that of the Jewish physician Mattathias, in 1290. Having cured the reigning sovereign, Shah Arghim, of a complaint which had baffled all the native doctors, he was rewarded with the choice of any post that he pleased at the Persian Court. The wary physician at once chose that of Minister of Finance, and acquitted himself so well in his new position as to become the Shah's adviser. The dignity of Vizier (Prime Minister) seemed already within his reach, when the dagger of a native fanatic ended at once his ambition and his life.

---

THE Royal Albert dock in London is 290 feet wide and a mile and a quarter long. The entire space is now lit by electricity. This is the greatest practical use to which the electric light has been put in England. Twenty-seven latticed poles, eighty feet high, have been erected, and in each pole is an electric lamp. The docks are now as light as day, and the loading and unloading of steamers goes on during the night as readily as in daylight. Four twenty-horse power engines supply the motive power. Many warehouses are lit by electricity in London, and the city calls for tenders for lighting with electricity the embankment the bridges, and many of the adjacent buildings.

